# Does post-operative radiotherapy improve the treatment outcomes of intracranial hemangiopericytoma? A retrospective study

**DOI:** 10.1186/s12885-021-08594-x

**Published:** 2021-08-12

**Authors:** Jianbiao Xiao, Lanwei Xu, Yi Ding, Wei Wang, Fen Chen, Yangshu Zhou, Fengjiao Zhang, Qiyuan Zhou, Xuehui Wu, Junpeng Li, Li Liang, Yee-Min Jen

**Affiliations:** 1 Department of Pathology, Nanfang Hospital and School of Basic Medical Science, Southern Medical University, 1838, Guangzhoudadao Rd, Guangzhou, Guangdong Province 510515 People’s Republic of China; 2 Guangdong Province Key Laboratory of Molecular Tumor Pathology, Guangzhou, 510515 Guangdong Province People’s Republic of China; 3 Department of Hand and Foot Surgery, Shandong Provincial Hospital Affiliated to Shandong First Medical University, Jinan, China; 4 Department of Radiation Oncology, Nanfang Hospital, Southern Medical University, Guangzhou, Guangdong Province China; 5 Department of Pathology, General Hospital of Southern Theater Command, People’s Liberation Army of China, Guangzhou, China; 6 Radiotherapy Center, Chenzhou No.1 People’s Hospital, Chenzhou, Hunan Province China; 7 Department of Pathology, Zhujiang Hospital, Southern Medical University, Guangzhou, Guangdong Province People’s Republic of China; 8 Department of Radiotherapy, Shanghai Concord Medical Cancer Center, Shanghai, China; 9 Department of Obstetrics and Gynecology, General Hospital of Southern Theater Command, People’s Liberation Army of China, Guangzhou, China; 10 Department of Radiation Oncology, Yee Zen General Hospital, 30, Yangxing North Rd, Yang Mei District, Tao Yuan City, Taiwan

**Keywords:** Intracranial hemangiopericytoma, Post-operative radiotherapy, stereotactic radiosurgery, Intensity- modulated radiotherapy

## Abstract

**Background:**

Intracranial hemangiopericytoma is a rare disease and surgery is the mainstay treatment. Although postoperative adjuvant radiotherapy is often used, there are no reports comparing different radiotherapy techniques. The purpose of this study is to analyze the impact of post-operative radiotherapy and different radiotherapy technique on the results in patients with intracranial hemangiopericytoma (HPC).

**Methods:**

We retrospectively reviewed 66 intracranial HPC patients treated between 1999 and 2019 including 29 with surgery followed by radiotherapy (11 with intensity-modulated radiotherapy (IMRT) and 18 with stereotactic radiosurgery (SRS)) and 37 with surgery alone. Chi-square test was used to compare the clinical characteristic between the groups. The Kaplan-Meier method was used to analyze overall survival (OS) and recurrence-free survival (RFS). Multivariate Cox proportional hazards models were used to examine prognostic factors of survival. We also underwent a matched-pair analysis by using the propensity score method.

**Results:**

The crude local control rates were 58.6% in the surgery plus post-operative radiotherapy group (PORT) and 67.6% in the surgery alone group (*p* = 0.453). In the subgroup analysis of the PORT patients, local controls were 72.7% in the IMRT group and 50% in the SRS group (*p* = 0.228). The median OS in the PORT and surgery groups were 122 months and 98 months, respectively (*p* = 0.169). The median RFS was 96 months in the PORT group and 72 months in the surgery alone group (*p* = 0.714). Regarding radiotherapy technique, the median OS and RFS of the SRS group were not significantly different from those in the IMRT group (*p* = 0.256, 0.960). The median RFS were 112 and 72 months for pathology grade II and III patients, respectively (*p* = 0.001). Propensity score matching did not change the observed results.

**Conclusion:**

In this retrospective analysis, PORT did not improve the local control rates nor the survivals. The local control rates after IMRT and SRS were similar even though the IMRT technique had a much higher biological dose compared with the SRS technique.

## Background

In 1942, Stout AP and Margaret RM described a new type of vascular tumor showing the characteristic formation of endothelial tubes and sprouts surrounded by a sheath of rounded and sometimes elongated cells. They believed that these cells were derived from the capillary pericytes and suggested that the tumors be called hemangiopericytoma (HPC) [1]. In 2014, WHO reclassified hemangiopericytoma as solitary fibrous tumor [2]. From its discovery until 1954, a total of 38 cases were reported. In 1954, Begg and Garret reported the first patient of primary cranial meningeal HPC [3]. The tumor histologically resembled both the soft tissue HPC previously described by Stout and Murray and the aggressive variant of angioblastic meningioma reported by Cushing and Eisenhardt [4]. Compared to extracranial HPC, intracranial HPC is less frequent and remains a rare entity, representing 0.4% of all primary central nervous system tumors [5]; meningioma is approximately 50 to 60 times more common than intracranial HPC [6–8]. It was reported that 1.6–2.5% of tumors diagnosed as meningiomas by neuroimaging were intracranial HPC [9, 10]. The histological origin of central nervous system HPC has been controversial for a long time, and it is now widely accepted that this tumor arises from the meningeal capillary pericytes. The current WHO classification includes HPC in the group of meningeal mesenchymal non-meningothelial tumors with uncertain malignant potential or borderline malignancy [11–13].

In a study of 191 HPC patients, the authors reported that at first local recurrence, patients who underwent repeated surgery survived longer than those patients who did not (median survival time, 53.0 months vs. 35.7 months; *P* = 0.028) [14]. In another report, postoperative radiotherapy (PORT) was shown to reduce local recurrence from 88% with surgery alone to 12.5% with PORT [15]. Kim et al. demonstrated that routine PORT with 50 to 60 Gy regardless of the resection margin and histology significantly improved the median time to local recurrence from 19.5 months in those without PORT to 80.5 months in patients with PORT (*p* = 0.0003) [16]. Some studies have suggested that complete surgical removal followed by PORT to the tumor bed is the best treatment policy for intracranial HPCs [9, 17, 18]. Others concluded that postoperative external beam radiotherapy (EBRT) to the tumor bed appears to delay recurrence [9, 17].

Although adjuvant radiotherapy after surgery has been frequently used in the treatment of HPC, there are no reports comparing conventionally fractionated EBRT and stereotactic radiosurgery (SRS) in HPC. The purpose of this study is to analyze the impact of PORT and different PORT techniques (conventionally fractionated EBRT versus SRS) on treatment efficacy of 66 patients with intracranial HPC.

## Methods

### Patients

This is a retrospective, observational study. The study protocol was approved by the hospital review board of the five participant hospitals. The medical charts and electronic databases of the hospitals were searched to identify patients diagnosed with primary intracranial HPC who had surgery as the first therapy between 1999 and 2019. Three patients who underwent initial surgery at other hospitals and were later treated for recurrent diseases at one of the five hospitals were also included. All of the patients had pathological confirmation of HPC. Information about their demographic data, pathology, surgery, PORT dose and technique, tumor control, survival status, and treatment-related side effects, were recorded. The pathology slides, when available, were reviewed by one of the authors to confirm the diagnosis.

One of the five participant hospitals used SRS routinely for their intracranial HPC patients. Patients from the other four hospitals were treated with conventionally fractionated intensity-modulated radiotherapy (IMRT) using linear accelerators. The patients were divided into surgery plus PORT group and surgery alone group; patients in the surgery plus PORT group were further separated into the IMRT group and the SRS group for subgroup analysis. All of the patients were evaluated clinically and radiologically with magnetic resonance imaging (MRI) scans with and without contrast. Follow-up MRI images were compared to the preoperative images by one of the authors and the tumor dimensions, if present, were measured in the axial, sagittal, and coronal planes.

### Statistical methods

The primary endpoint is local tumor control, and secondary endpoints include overall and recurrence-free survivals. All of the statistical analyses were performed by using SPSS v.19. (SPSS Inc., Chicago, IL, USA). Mann-Whitney U-tests (two-tailed) were used to analyze the differences in continuous variables. Fisher’s exact test (two-tailed) was used to examine the differences in categorical variables. Chi-squared test was conducted to compare the differences of clinical characteristics between groups. Kaplan-Meier method was applied to obtain local control, overall survival (OS) and recurrence-free survival (RFS). The outcomes were compared between those who receive and did not receive PORT and also between the IMRT and the SRS groups. Univariate and multivariate Cox proportional hazards models were used to search for potential prognostic variables including age (> 50 years), sex, tumor location, tumor resection, pathology grade and radiation therapy.

To reduce the patient heterogeneity inherent in a retrospective study between the two groups, the R package “MatchIt” was used to conduct a propensity score matching. Treatment outcome analyses were repeated after 29 from the 37 surgery alone patients were matched to the 29 patients with postoperative radiotherapy. Variables matched included age, sex, tumor location, post-operative radiotherapy technique, resection extent and pathology grade.

## Results

### Patients and radiotherapy techniques

A total of 66 patients diagnosed with intracranial HPC were collected (Table 1). There were 35 males and 31 females with 83.3% having supratentorial lesions. Gross tumor resection (GTR) was conducted in 61 (92.4%) and subtotal tumor resection (STR) in 5 (7.6%) patients. Grade II HPCs were diagnosed in 35 (53%) patients, and grade III HPCs (anaplastic HPC) were diagnosed in 31 (47%) patients. Twenty-nine (43.9%) patients had surgery followed by radiotherapy while 37 (56.1%) had surgery alone. The differences in patient characteristics between the two groups with and without PORT were not statistically significant (Table 1). The median follow-up time after operation in all patients was 50.5 months (range, 2–153); it was 57 and 47 months in the surgery plus PORT and the surgery alone groups, respectively. For patients alive at the time of analysis, the median follow-up time was 66 months for the surgery plus PORT group and 58 months for the surgery alone group.

**Table 1:** Characteristics of 66 patients with intracranial hemangiopericytoma by the treatment types

	All patients N (%)	S + PORT (29 patients) N (%)	S (37 patients) N (%)	*P* value
Age in years				0.892
> 50	29(43.9%)	13(44.8%)	16(43.2%)	
< 50	37(56.1%)	16(55.2%)	21(56.8%)	
Sex				0.493
Male	35(53%)	14(48.3%)	21(56.8%)	
Female	31(47%)	15(51.7%)	16(43.2%)	
Tumor location				0.579
Supratentorial	55(83.3%)	25(86.2%)	30(81.1%)	
Infratentorial	11(16.7%)	4(13.8%)	7(18.9%)	
Pathology grade				0.493
Grade II	35(53%)	14(48.3%)	21(56.8%)	
Grade III	31(47%)	15(51.7%)	16(43.2%)	
Extent of resection				0.452
GTR	61(92.4%)	26(89.7%)	35(94.6%)	
STR	5(7.6%)	3(10.3%)	2(5.4%)	
Recurrence				0.453
Yes	24(36.4%)	12(41.4%)	12(12.4%)	
No	42(63.6%)	17(58.6%)	25(67.6%)	

*PORT* post-operative radiotherapy, *S* surgery, *GTR* gross total resection, *STR* subtotal resection

**Fig. 1:**
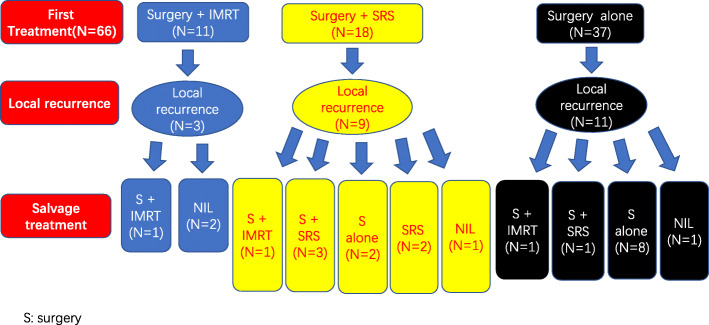
A diagram showing the initial patient treatments, local recurrence and salvage therapy after local recurrence. Twenty-nine (43.9%) patients had surgery followed by post-operative radiotherapy (PORT) while thirty-seven (56.1%) had surgery alone. Of the 29 patients with PORT after operation, 11 received intensity-modulated radiotherapy and 18 had stereotactic radiosurgery (SRS) (12 had gamma knife SRS and 6 had linac-based SRS). There were 12 local recurrences out of the 29 patients with surgery plus PORT, and 11 of the 37 surgery alone patients

Of the 29 patients with PORT after operation, 11 received IMRT and 18 had SRS (12 had gamma knife SRS and 6 had linac-based SRS) (Table 2). The clinical characteristics were not significantly different between the surgery and PORT groups; they were also similar between the PORT-IMRT and the PORT-SRS groups including the extent of tumor resection and the tumor grade. Eleven patients received fractionated IMRT with a median fraction number of 30 and a median prescription dose of 60 Gy (range 50–60 Gy). IMRT was delivered with 6 MV photons from linear accelerators (Varian Trilogy and Clinac IX; Elekta Synergy). Clinical target volume (CTV) was defined as the tumor cavity and/or the residual mass plus a 5–10 mm margin. An additional 3–5 mm was added to the CTV for planning target volume. Eighteen patients underwent gamma knife SRS with a single dose of 14–16 Gy at the margin of tumor (12 had gamma knife SRS and 6 had X ray SRS) (Gamma Knife, Elekta, Perfection; Varian Clinac 23ES). Table 3 presents the characteristics of the PORT patients and the surgery alone cohort matched with the PORT patients. The differences between the two groups were not improved after propensity score matching. At the time of the study cutoff day, 24 of the 29 patients in the PORT group and 25 of 37 patients in the surgery alone group were alive. Figure 1 shows the study framework including the initial patient treatments, local recurrence and salvage therapy after local recurrence.

**Table 2:** Characteristics of 29 patients with intracranial hemangiopericytoma receiving post-operative radiotherapy divided by radiotherapy technique

	All patients N (%)	IMRT (11 patients) N (%)	SRS (18 patients) N (%)	*P* value
Age in years				0.074*
> 50	13(44.8%)	7(63.6%)	6(33.3%)	
< 50	16(55.2%)	4(36.4%)	12(66.7%)	
Sex				0.316
Male	14(48.3%)	4(36.4%)	8(55.6%)	
Female	15(51.7%)	7(63.6%)	8(44.4%)	
Tumor location				1.000
Supratentorial	25(84.2%)	10(90.9%)	15(83.3%)	
Infratentorial	4(15.8%)	1(9.1%)	3(16.7%)	
Pathology grade				0.316
Grade II	14(48.3%)	4(36.4%)	10(55.6%)	
Grade III	15(51.7%)	7(63.6%)	8(44.4%)	
Extent of resection				0.862
GTR	26(89.7%)	10(90.9%)	16(88.9%)	
STR	3(10.3%)	1(9.1%)	2(11.1%)	
Recurrence				0.228
Yes		3(27.4%)	9(50%)	
No		8(72.7%)	9(50%)	

*RT* radiotherapy, *IMRT* intensity-modulated radiotherapy, *SRS* stereotactic radiosurgery, *GTR* gross total resection, *STR* subtotal resection

*P values are statistically significant

**Table 3:** Characteristics of 58 propensity score-matched patients with intracranial hemangiopericytoma by the treatment types

	All patients N (%)	S + PORT (29 patients) N (%)	S (29 patients) N (%)	*P* value
Age in years				0.594
> 50	24(41.4%)	13(44.8%)	11(37.9%)	
< 50	34(58.6%)	16(55.2%)	18(62.1%)	
Sex				0.110
Male	34(58.6%)	14(48.3%)	20(69.0%)	
Female	24(41.4%)	15(51.7%)	9(31.0%)	
Tumor location				0.717
Supratentorial	49(84.5%)	25(86.2%)	24(82.8%)	
Infratentorial	9(15.5%)	4(13.8%)	5(17.2%)	
Pathology grade				0.110
Grade II	34(58.6%)	14(48.3%)	20(69.0%)	
Grade III	24(41.4%)	15(51.7%)	9(31.0%)	
Extent of resection				0.075
GTR	55(94.8%)	26(89.7%)	29(100.0%)	
STR	3(5.2%)	3(10.3%)	0(0.0%)	
Recurrence				0.269
Yes	20(34.5%)	12(41.4%)	8(27.6%)	
No	38(65.5%)	17(58.6%)	21(72.4%)	

*PORT* post-operative radiotherapy, *S* surgery, *GTR* gross total resection, *STR* subtotal resection

### Histological findings

**Fig. 2:**
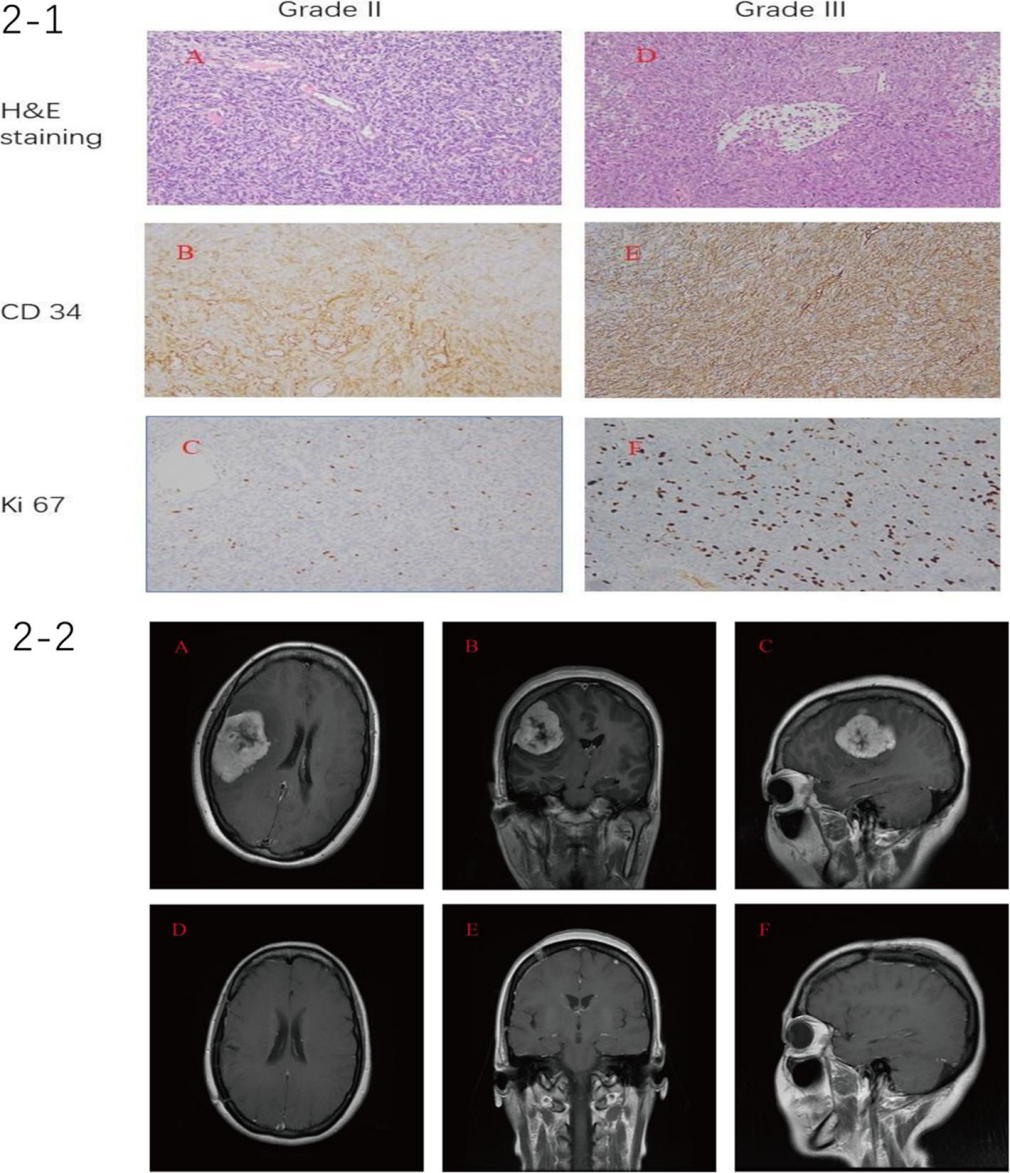
Figure (2-1). **A, B** and **C**: Microphotographs of the histologic images of a grade II intracranial hemangiopericytoma patient (magnification × 200). **A**, Hematoxylin & Eosin staining demonstrated an extensively vascularized and cellular tumor; **B**, Immunohistochemical staining showed a strong positivity for CD34; **C**, immunohistochemical staining showed a 5% positivity for Ki67; **D, E** and **F**: Microphotographs of the histologic images of a grade III intracranial hemangiopericytoma patient (magnification × 200). **D**, Hematoxylin & Eosin staining demonstrated an extensively vascularized and cellular tumor; **E**, Immunohistochemical staining showed a strong positivity for CD34; **F**, Immunohistochemical staining showed a 10% positivity for Ki67. Compared with grade II HPC, the grade III tumor showed a higher percentage of ki67 positivity and more prominent nuclear fission and cell morphology heterogeneity. Figure (2-2). Magnetic resonance imaging (MRI) of a grade II intracranial hemangiopericytoma patient without recurrence after gross total resection. **A-C**: Preoperative T1-weighted MRI scans with contrast: **A** axial, **B** coronal and **C** sagittal images, showing an enhanced lesion at the right temporal lobe with central necrosis. **D-F** Postoperative T1-weighted MRI scans with contrast: **D** axial, **E**, coronal, and **F**, sagittal images, showing a complete tumor removal. The patient was recurrence-free 22 months after surgery. His histologic images were shown in Fig. 2–1

The pathological specimens with Hematoxylin & Eosin (H&E) staining of all the 66 patients showed an extensively vascularized and cellular tumor. These tumors showed compact and uniform cells with a large number of small vascular cavities and compact reticular fibers. Immunohistochemical (IHC) staining showed a strong positivity for CD34. The percentage of ki67 positivity was lower in grade II HPC compared with grade III tumors; the median percentage of positive staining for Ki67 was 2% (range 1–5%) in grade II HPC and 12% in grade III HPC (range 10–16%). There were more prominent nuclear fission and cell morphology heterogeneity in the higher grade HPC. IHC was negative for PR, S-100 and EM (Fig. 2).

### Imaging findings

All of the patients had MRI before and after operation. After craniotomy, MRI was repeated at 3–6-month intervals in the first 3 years with and without contrast. The pre-contrast MRI showed a hypointense lesion on T1-weighted images (WI) and a heterogeneously hyperintense lesion on T2WI. A flow void signal was present in most tumor images, and cystic tumor necrosis and the dural tail sign were also very common. Contrast-enhanced MRI often showed markedly and heterogeneously enhanced lesions. Fig. 2 showed one of the HPC patient’ MRI images before and after operation.

### Local control and survival

**Fig. 3:**
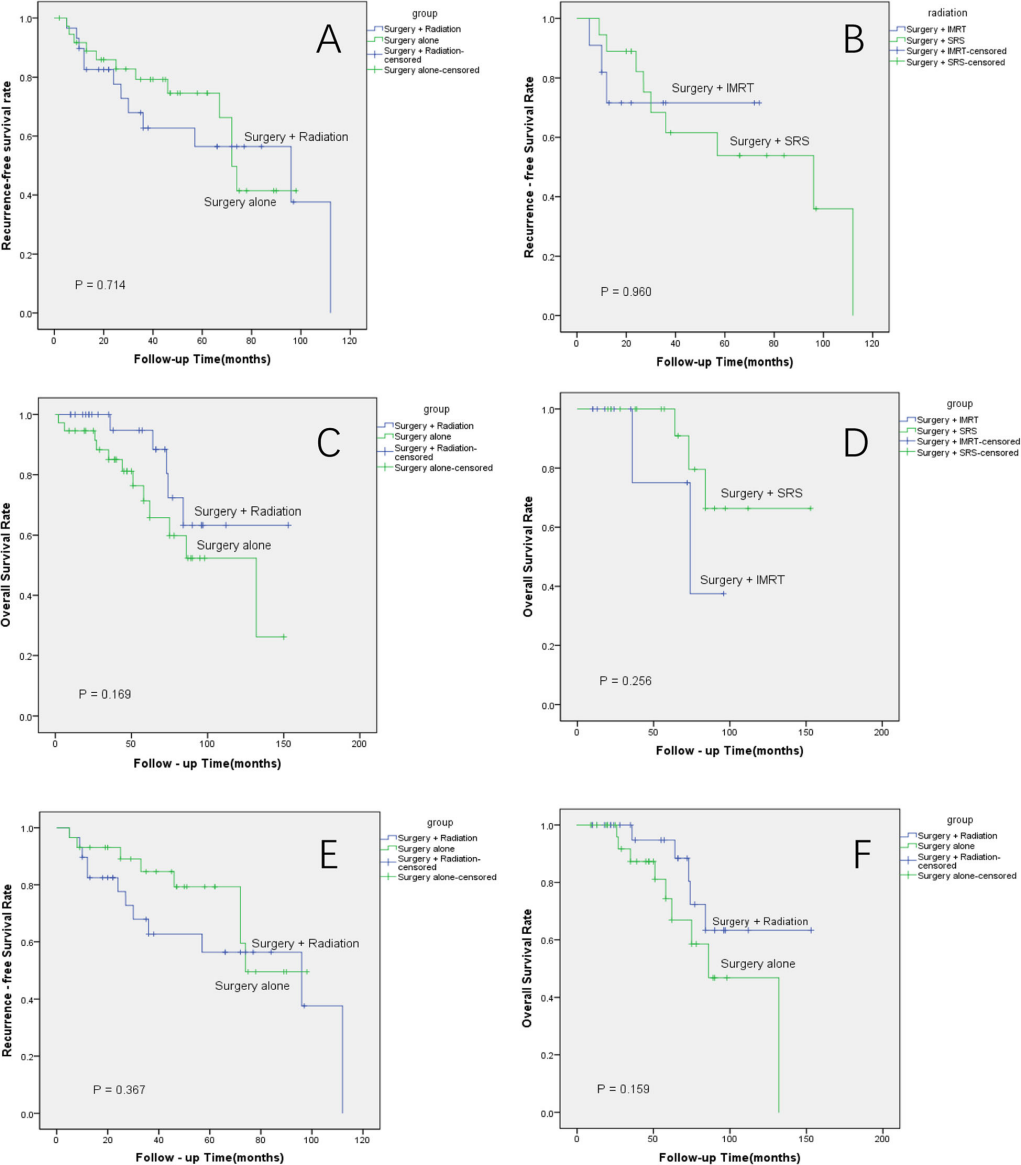
**A** Kaplan-Meier estimates of the local recurrence-free survival (RFS) curves in intracranial hemangiopericytoma patients. Postoperative radiotherapy (PORT) did not increase the RFS (*p* = 0.714). **B** Kaplan-Meier estimates of the local RFS curves in intracranial hemangiopericytoma patients with PORT. The RFS of the two groups with different radiotherapy techniques were similar (*p* = 0.960). **C** Kaplan-Meier estimates of the overall survival (OS) curves of intracranial hemangiopericytoma patients. There were no differences between the patients with and without PORT (*p* = 0.169). **D** Kaplan-Meier estimates of the OS curves in intracranial hemangiopericytoma patients with PORT. The OS of the two groups were similar (*p* = 0.256). **E** Kaplan-Meier estimates of the recurrence-free survival curves of propensity score-matched intracranial hemangiopericytoma patients (surgery plus radiation and surgery alone groups). Postoperative radiotherapy did not increase the RFS (*p* = 0.367). **F** Kaplan-Meier estimates of the overall survival curves of propensity score-matched intracranial hemangiopericytoma patients (surgery plus radiation and surgery alone groups). There were no differences between the patients with and without PORT (*p* = 0.159)

The crude local control rates were 58.6% in the surgery plus PORT group and 67.6% in the surgery alone group; Fig. 3A shows the local recurrence-free survival of both groups (*p* = 0.714). In the subgroup analysis of the PORT patients, they were 72.7% (8/11) in the IMRT group and 50% (9/18) in the SRS; the local-recurrence free survivals are shown in Fig. 3B (*p* = 0.960).

The median RFS of grade II and III patients were 112 and 72 months, respectively (*p* = 0.001). Salvage surgery with or without PORT was conducted for most patients with local recurrence. The 5-year RFS rates in the surgery plus PORT group and surgery alone group were 56.4 and 74.6%, respectively.

The median OS in the surgery plus PORT and surgery alone groups were 122 months and 98 months, respectively (*p* = 0.169) (Fig. 3C). The median OS in the SRS and IMRT groups were 127 months and 73 months (*p* = 0.256) (Fig. 3D). The 5-year OS rates in the PORT and surgery alone groups were 75 and 90.9%, respectively.

With the propensity score matching, the crude local control rates were 58.6% in the surgery plus PORT group and 72.4% in the surgery alone group. Figure 3E displays the local recurrence-free survival of both groups (*p* = 0.367). For the matched-pair cohort, the median OS in the surgery plus PORT and surgery alone groups were 122 months and 93 months, respectively (*p* = 0.159) (Fig. 3F); the 5-year overall survival were 51.7% for the surgery plus PORT and 34.5% for the surgery alone (*p* = 0.289). The 5-year RFS were 65.5% for the surgery plus PORT and 82.8% for the surgery alone group (*p* = 0.230).

### Prognostic factors of OS and RFS

Age > 50 years is the only prognostic factor for OS by both the univariate (*p* = 0.024) and multivariate (*p* = 0.029) Cox regression analyses (Table 4). The median OS time is 84 months in the older group (age > 50 years) and 122 months in the younger group (age < 50 years) (*p* = 0.018). The median RFS time is 72 months in the older group (age > 50 years) and 96 months in the younger group (age < 50 years) (*p* = 0.100). In Table 4, the 95% CI for location and resection were very large. The reason is that all the 11 infratentorial patients and all the STR patients were alive and therefore censored at the time of univariate and multivariate analysis, making it impossible to obtain a statistical result.

**Table 4:** Univariate and Multivariate Analysis of Factors Associated with Overall Survival in Patients of Intracranial Hemangiopericytoma

Variable	Univariate Analysis		Multivariate Analysis	
	OS		OS	
	*P*	95% CI	*p*	95% CI
Age	0.024^*^	0.121–0.863	0.029^*^	0.103–0.883
Sex	0.282	0.219–1.558	0.573	0.406–5.096
Location	0.210	0.000–6.567	0.963	0.000
Resection (GTR)	0.395	0.000–61.834	0.971	0.000
Recurrence	0.282	0.203–1.591	0.348	0.136–2.022
Pathology grade	0.303	0.618–4.712	0.898	0.335–3.478
Radiation (Yes)	0.215	0.681–5.533	0.133	0.114–1.333

*OS* Overall survival, *CI* confidence interval, *GTR* gross total resection

*P values are statistically significant

The median RFS of patients with grade II and III were 112 and 72, respectively (*p* = 0.001). Pathological grade is the only prognostic factor for RFS by both the univariate (*p* = 0.003) and multivariate (*p* = 0.005) Cox regression analyses (Table 5).

**Table 5:** Univariate and Multivariate Analysis of Factors Associated with Recurrence-free Survival in Patients of Intracranial Hemangiopericytoma

Variable	Univariate Analysis		Multivariate Analysis	
	RFS		RFS	
	*P*	95% CI	*p*	95% CI
Age	0.107	0.208–1.166	0.083	0.173–1.114
Sex	0.231	0.248–1.400	0.302	0.229–1.580
Location	0.787	0.293–2.538	0.518	0.175–2.409
Resection (GTR)	0.103	0.832–7.404	0.238	0.591–8.314
Pathology grade	0.003^*^	1.639–10.663	0.005^*^	1.495–9.813
Radiation (Yes)	0.716	0.375–1.961	0.781	0.463–2.783

*RFS* Recurrence free survival, *CI* confidence interval, *GTR* gross total resection

*P values < 0.5

## Discussion

Intracranial HPC is a rare disease, and it is also rare in the literature for any single study having a large case number and a satisfactory length of follow-up. In our present report on 66 patients, some of our findings concur with the previously published results in the literature, but some do not. Impact of the extent of tumor resection have been examined by several studies, and complete tumor resection was shown to play a pivotal role for both local control and survival [7, 12–23]. In our study, however, GTR did not affect the treatment results. This may be explained by that 90% of our patients had GTR and thus making it difficult to detect a difference statistically when compared with the small number of patients with residual tumor.

Most studies investigating the effect of PORT in intracranial HPC were based on single-center analysis with a limited patient number, and the results were often contradictory. Meta-analyses and studies based on accumulated database have been conducted to overcome the problems of small case series, but their results were also inconsistent [24–26]. Although the role of PORT in the GTR patients is not clear, the general consensus is that PORT is beneficial for patients undergoing STR. Some studies found that PORT following STR improves both RFS and OS compared with STR alone [8, 25–28], and the others reported that PORT following GTR may also prolong OS [24, 26, 28, 29] or improve local control [22, 30]. Contrary to the above, some authors have reported that PORT after GTR has no impact on survival [6, 25, 31] or that PORT should not be used except for patients with recurrences [32, 33]. In our study, we have found that PORT after surgery has no significant impact on the overall and disease-free survivals. This is in line with the results from Lee et al. who underwent an analysis of practice pattern in the US for intracranial HPC and the PORT effect on its survival. They obtained data of 588 cases from the cancer registry, of which 323 (54.9%) received postoperative radiation. The 5-year overall survival for those receiving PORT 77.1%, not significantly different from the 83.8% for those who did not (*p* = 0.14). Postoperative radiation was not prognostic for survival on multivariable analysis [34]. This observation was not altered after propensity score matching.

SRS has been used for the patients with residual or recurrent intracranial HPCs [18, 32, 35–39]. It was reported that postoperative SRS resulted in a better local tumor control in intracranial HPC patients [18]. In our subgroup analysis, patients with SRS had a similar OS to the patients with IMRT. The biologically effective dose (BED) of the SRS and IMRT groups for HPC with an α/β ratio of 10 Gy was about 33.6–41.6 Gy_10_ and 72 Gy_10_, respectively. The biological effectiveness of the IMRT technique was much higher than the SRS method, yet resulting in no higher local control. The reason is not clear why a higher BED did not result in a higher local effect. However, considering that SRS may be able to minimize the radiation to the adjacent tissues due to the high-precision delivery of radiation to HPC with a steep radiation dose gradient, it is a reasonable PORT option for HPC patients [32, 40]. There is no consensus on the optimal radiation dose for single-fraction SRS for intracranial HPC. Some centers have reported an improved local control at higher tumor margin doses, ranging from 14 to 17 Gy [41, 42], but other studies using tumor margin doses exceeding 20 Gy did not show an improved local control [43, 44].

From our analysis, PORT does not seem to improve local control, RFS and overall survival. Yet caution in interpretating the present findings is needed due to the limitation of this study that includes the small sample size and its retrospective nature. To examine the exact impact of PORT on local control and survival and the different effects between IMRT and SRS as a PORT option requires a multi-center randomized trial with a larger sample size.

## Conclusion

Before the arrival of more convincing data from a randomized trial, we have to rely on results from suboptimal studies like this retrospective analysis, with prudent judgement. Although PORT did not improve the local control rates nor the survivals in this retrospective analysis, in view of the limitations of the present report and the existing conflicting results, PORT may still be considered in HPC. The finding that local control rates after IMRT and SRS were similar suggests that both radiotherapy techniques are viable options.

## Data Availability

The datasets of the study are available on request to the correspondent authors.

## Abbreviations

IMRT: Intensity-modulated radiotherapy
SRS: Stereotactic radiosurgery
OS: Overall survival
RFS: Recurrence-free survival
PORT: Post-operative radiotherapy
HPC: Hemangiopericytoma
WHO: World Health Organization
EBRT: External beam radiotherapy
GTR: Gross tumor resection
STR: Subtotal tumor resection
CTV: Clinical target volume
H&E: Hematoxylin & Eosin
IHC: Immunohistochemical
